# Deep Brain Stimulation for Parkinson's Disease

**DOI:** 10.14789/jmj.JMJ22-0041-R

**Published:** 2023-02-22

**Authors:** ATSUSHI UMEMURA

**Affiliations:** 1 Department of Research and Therapeutics for Movement Disorders, Juntendo University Graduate School of Medicine, Tokyo, Japan; 1 Department of Research and Therapeutics for Movement Disorders, Juntendo University Graduate School of Medicine, Tokyo, Japan; 2 Department of Neurosurgery, Juntendo University School of Medicine, Tokyo, Japan; 2 Department of Neurosurgery, Juntendo University School of Medicine, Tokyo, Japan

**Keywords:** Parkinson's disease, deep brain stimulation, subthalamic nucleus

## Abstract

There is a long history of surgical treatment for Parkinson's disease (PD). Currently, deep brain stimulation (DBS) has been performed as promising treatment option for medically refractory PD. DBS is an adjustable and reversible treatment using implanted medical devices to deliver electrical stimulation to precisely targeted areas of the brain. DBS modulates neurological function of the target region. The most common target for PD is the subthalamic nucleus (STN). DBS is particularly indicated for patients suffering from motor complications of dopaminergic medication such as fluctuations and dyskinesia. Although there is currently no curative treatment for PD, a combination of medical treatment and DBS provide long-term relief of motor symptoms. In this review, I introduce history, mechanism, indication, clinical outcome, complication, long term outcome, timing of surgery, surgical procedure, and current new technology concerning DBS for PD.

## Introduction

Parkinson's disease (PD) is a progressive nervous disorder caused by degeneration of dopamine-producing cells in the substantia nigra. The main symptoms are movement-related, including tremor, rigidity, bradykinesia, postural instability, gait disturbance, and so on. Other symptoms include autonomic, sensory, psychiatric and cognitive problem. Although there is currently no curative treatment for PD, motor symptoms of PD are initially treated with dopaminergic medications such as levodopa and dopamine agonists. In addition to medical treatment, there is a long history of surgical treatment for Parkinson's disease (PD)^1)^. After pioneering trials and errors, the current primary surgical treatment for PD is deep brain stimulation (DBS). To date, more than 170,000 patients worldwide have undergone DBS. DBS is a promising treatment option for patients with medically refractory PD.

## History of surgical treatment for Parkinson's disease

James Parkinson published “An essay on the shaking palsy” in 1817. However, the etiology and cure for this intractable disease long remained unknown. The concept of the extrapyramidal tract was proposed in the 1920s and direct surgery to the basal ganglia was attempted. In 1947, Spiegel and Wycis developed a stereotactic frame for humans, enabling less invasive surgery to the extrapyramidal tract^2)^. Stereotactic pallidotomy or thalamotomy was subsequently developed for the treatment of PD. However, the use of surgical treatment rapidly declined after the introduction of levodopa in 1969. Dopamine replacement therapy became a mainstay of the treatment of PD. However, some patients suffered from motor complications of dopaminergic medication such as fluctuation or dyskinesia. In 1992, Laitinen revived pallidotomy for patients with motor complications from levodopa^3)^.

In 1983, Langston found 1-methyl-4-phenyl- 1,2,3,6-tetrahydropyridine (MPTP) as a neurotoxin causing PD^4)^. Consequently, an animal model for PD using MPTP was developed and the pathophysiology of PD was clarified in detail. In 1989, Albin demonstrated the functional anatomy of the basal ganglia related to the pathophysiology of movement disorders^5)^. Shortly after, Bergman demonstrated that motor symptoms of MPTP-treated monkeys were dramatically improved by lesioning of the subthalamic nucleus (STN)^6)^. In 1993, Benabid et al developed deep brain stimulation (DBS) of the STN, a monumental work of surgical treatment of PD^7)^. Subsequently, DBS of the globus pallidus internus (GPi) was introduced as an another treatment option.

## DBS and its mechanism

**Figure 1:**
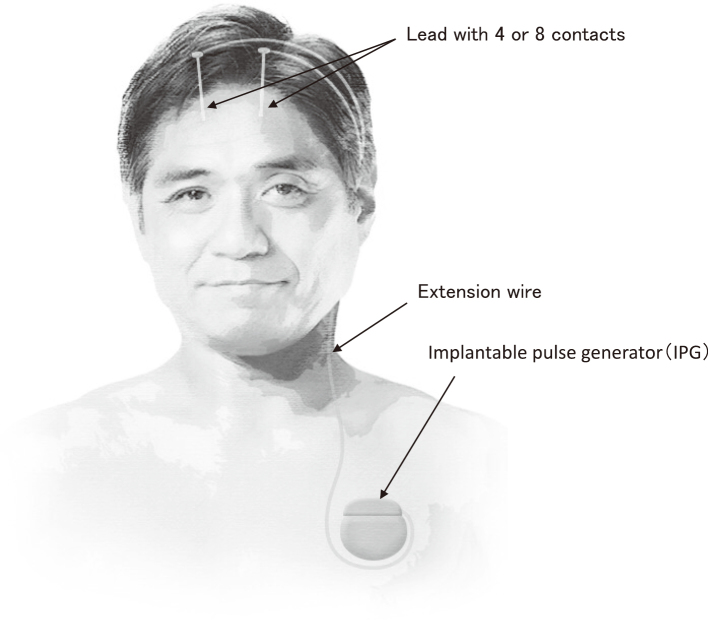
Outline of DBS system

DBS is a surgical treatment involving the implantation of medical devices to deliver electrical stimulation to precisely targeted areas of the brain. Expectation of DBS is based on functional alteration in the target area. The DBS system consist of three components: the implantable pulse generator (IPG), the lead with four or eight contacts, and the extension wire (Figure 1). DBS is an adjustable and reversible treatment. The IPG can deliver pulses with three variable parameters. Frequency (2-250 Hz), width (20-450 µsec), amplitude (0-20 mA), and the selection of the stimulating contact can be set by wireless telemetry.

**Figure 2:**
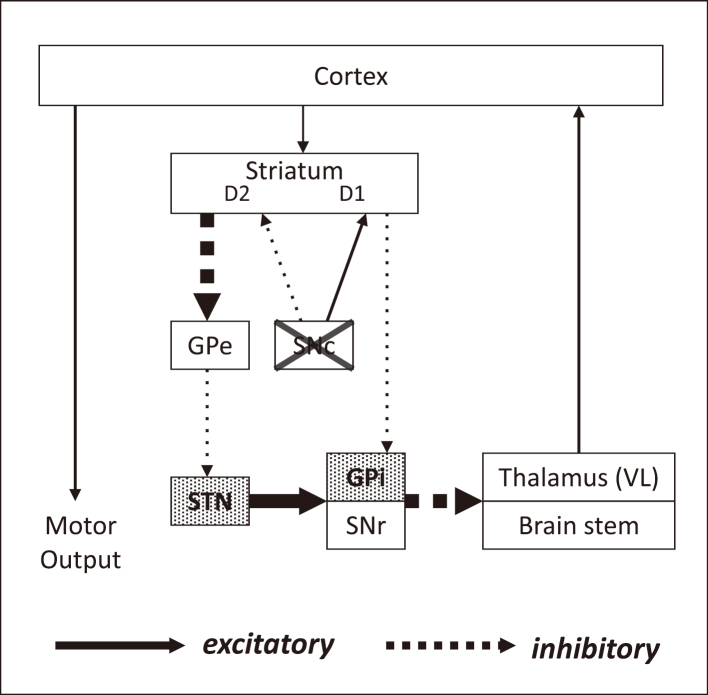
Pathophysiology of PD within the basal ganglia-thalamo-cortical circuit

Surgical treatment is based on the following functional alteration within the basal ganglia-thalamo-cortical circuit (Figure 2). In PD, increased excitatory activity of the STN caused by depletion of dopamine in substantia nigra pars compacta (SNc) abnormally activates the GPi which inhibit activity of the thalamus and thalamocortical neurons. The reduced thalamic and cortical activity account for the hypokinetic symptoms of PD such as rigidity and akinesia. Therefore, reducing the overactivity of STN or GPi through ablative procedure or DBS might have a considerable clinical effect in PD. DBS seems to produce a functional lesion in the brain and reduces activity in the focal area as well as ablative procedure. However, the true mechanism of the action in DBS is not well understood.

## Indication of DBS for PD

Currently, there is no curative treatment for PD. First choice of treatment for PD is medical treatment. Therefore, what is required for surgical treatment in PD is to obtain improvement of symptoms that are difficult to improve with medical treatment, or to solve problems caused by medication. Symptoms that are difficult to improve with medical treatment include some tremors and axial symptoms. On the other hand, problems caused by medication include motor complications such as fluctuation and dyskinesia, and side effects of medication such as hallucinations, delusions, and impulse control disorders.

In considering indications for DBS^8)^, a correct diagnosis of idiopathic PD is essential. A good response to levodopa is a good indicator of a correct diagnosis of PD. Atypical parkinsonism or secondary PD are not indications for DBS. The most appropriate surgical candidate for DBS is a patient who suffers from the motor complications of dopaminergic medications such as fluctuation and dyskinesia. A patient who suffers from disabling tremor despite optimal medical treatment is also a good candidate for DBS. Furthermore, patients with dementia or active psychiatric issues are not indicated for DBS. Ideally, the patient should also be young (i.e., less than 70 years of age), although carefully-selected older patients can also be candidate. Some experts recommend excluding patients based on a mini-mental state examination cutoff score of 24.

As described below, STN-DBS can reduce the dose of antiparkinsonian dopaminergic medication with improved motor function. Therefore, it is indicated for patients suffering from medication-induced psychotic symptoms such as hallucinations and delusions^9)^.

## Clinical outcomes

The theoretical target of DBS based on the pathophysiology of PD is the STN or the GPi. An early comparative study revealed the superiority of STN-DBS in improvement of motor scores in the medication-off period and reduction of dopaminergic medication^10)^. Consequently, the STN has long been the most common target of DBS for PD.

STN-DBS results in a significant reduction in the Unified Parkinson's Disease Rating Scale (UPDRS) motor score in the medication-off state but does not alter the score in the medication-on state. STN-DBS effectively improves levodopa-responsive symptoms of PD and significantly reduces dyskinesia, motor fluctuation, and the dose of dopaminergic medication. Several controlled randomized studies demonstrated that STN-DBS yielded better outcomes in motor function and quality of life (QOL) than medical treatment alone for advanced PD patients^11-13)^.

According to a meta-analysis of early outcomes, STN-DBS improves UPDRS III motor scores in the medication-off state by 52% and UPDRS II activities of daily living (ADL) score by 50%. STN-DBS also reduces dyskinesia by 69%, the daily off period by 68%, and the dose of dopaminergic medication by 56%. Average improvement in quality of life (QOL) using PDQ-39 is 35%^14)^. As a result, STN- DBS provides a second honeymoon period for patients suffering from the motor complications of dopaminergic medication.

Postural abnormality such as camptocormia or Pisa syndrome are one of the most difficult condition to treat. Postural abnormality could be corrected by DBS in some patient although the long-term benefit is limited^15)^. Early introduction of DBS after onset of postural abnormality is beneficial.

The effect of STN-DBS on impulse control disorders (ICD) and dopamine dysregulation syndrome (DDS) is controversial^16)^. Preexisting ICD or DDS is improved by considerable reduction of dopaminergic medication in some patients.

In this way, STN-DBS is currently the most effective surgical treatment for advanced PD because it has an overwhelming effect on motor symptoms and can solve many medication-induced problems by reducing the dose of dopaminergic medication.

## Complications of DBS

A significant incidence of adverse effects associated with DBS in PD has been reported^17)^. Most are mild and transient, but serious morbidity is also reported. According to a large study (1,183 patients)^18)^, the mortality rate during the first 30 postoperative days after stereotactic surgery is 0.4% and the permanent surgical morbidity rate is 1%. The morbidity is mainly caused by intracerebral hemorrhage (ICH) (2.2%). An analysis of adverse events in published data revealed that common surgery-related complications included 2.0% with symptomatic ICH and 2.0% with infections in 928 STN-DBS cases^19)^.

The neuropsychological aspects of STN-DBS have recently received considerable attention and many studies concerning neuropsychological outcome after STN-DBS have been performed^20)^. Mood changes including hypomania or depression are common adverse effects in patients treated with STN-DBS. Most are usually transient in the immediate postoperative period. The spread of stimulation to the limbic STN seems to be a cause of altered mood states^21)^. On the other hand, depression or apathy occurring several months after surgery often coincides with excessive reduction of dopaminergic medication, and is generally improved by increasing the dose.

There are many studies on cognitive outcomes after STN-DBS. Most concluded that STN-DBS is relatively safe from a cognitive perspective despite mild cognitive morbidity. A meta-analysis of cognitive sequelae revealed small but significant declines in executive function and verbal learning and memory, and moderate declines in both semantic and phonemic verbal fluency after STN DBS^22)^.

Several factors are considered to contribute to cognitive changes after STN-DBS. As the STN has widespread connections with basal ganglia and the prefrontal cortex^21)^, the direct effect of stimulation may contribute to cognitive changes. Furthermore, the impact of surgical intervention or drastic postoperative reduction of dopaminergic medication may cause cognitive decline^23)^.

## Long-term outcomes

There are many studies of long-term (more than 5 years) outcomes of STN-DBS^24)^. In most studies, effects of STN-DBS were mostly preserved even 5 years after surgery. For each symptom, improvements in cardinal motor symptoms such as tremor, rigidity, and bradykinesia are well maintained 5 years after surgery. However, axial symptoms affecting speech, gait, and postural instability progressively worsened. These symptoms are refractory to both medication and DBS. The symptoms of gait disturbance or postural instability seem to be mediated by nondopaminergic mechanisms. STN-DBS improves only the dopamine-mediated motor symptoms. Therefore, the aggravation of axial symptoms reflects the progression of PD itself.

There have been a few reports on long-term outcomes of STN-DBS of greater than 5 years^25)^. According to these reports, not only deterioration of axial motor symptoms but also cognitive decline affect worsening of ADL.

As for survival of patients with PD, Ngoga et al demonstrated that patients with STN-DBS have significantly longer survival than those who are treated only by medication. STN-DBS markedly reduces the death rate related to respiratory complications, such as pneumonia^26)^.

## Timing of surgery

**Figure 3:**
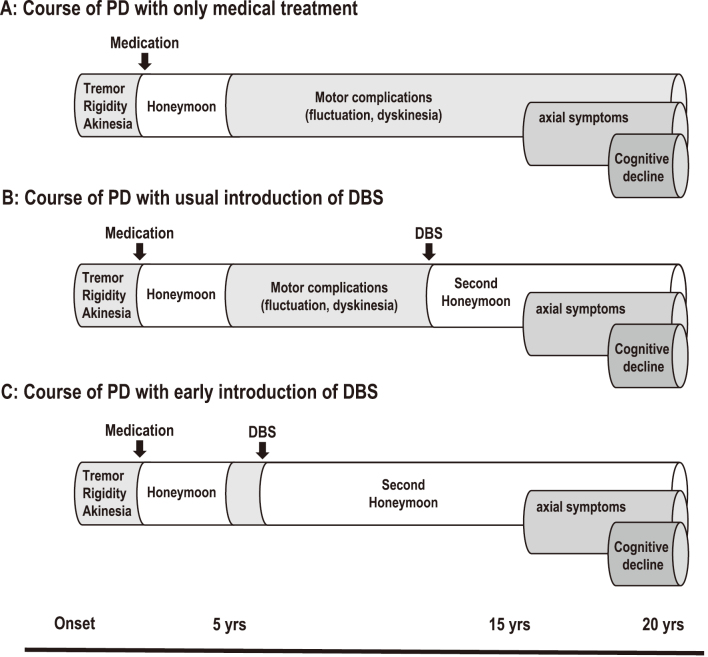
Timing of DBS in long-term course of PD

The timing of DBS surgery is one of current topics. The general course of PD with only medical treatment is shown in Figure 3A. After onset, patients remain well with medication for several years (honeymoon period). However, most patients subsequently suffer from motor complications of dopaminergic medication such as fluctuation and dyskinesia. In the advanced stage, treatment-resistant axial symptoms and cognitive decline appear. Until now, DBS has been considered as a last resort after medical treatment, and was usually introduced in the late phase of motor complications (Figure 3B). In this situation, patients could achieve a second honeymoon period after DBS, but treatment-resistant axial symptoms appeared in several years. Currently, early introduction of STN-DBS is recommended based on new evidence (EARLYSTIM study)^27)^. This study demonstrated that STN-DBS also improved QOL and motor function in PD with early motor complications. In this situation, the second honeymoon period will be much longer (Figure 3C).

## Surgical procedure

The surgical procedure for STN-DBS varies among centers^28)^. There is some controversy about surgical aspects of DBS. Currently, DBS leads are implanted into the target area stereotactically under magnetic resonance imaging (MRI) guidance with physiological refinement by microelectrode recording (MER) under local anesthesia in most centers. DBS surgery proceeds logically, and although a certain amount of experience is required to make various decisions during surgery, anyone can complete it by performing several steps steadily.

As recent progress in MRI technology has enabled direct visualization of the STN, some groups avoid using MER for placement of the DBS lead. They insist that MER may increase the risk of ICH. The combination of MER and hypertension will definitely increase the incidence of bleeding. However physiological refinement by MER is the gold standard for identifying the STN and its borders. The optimal region for STN stimulation might be missed due to individual anatomical variations or intraoperative brain shift. In our own series, about 20% of cases required two or more trajectories to obtain sufficient activity of STN by MER^29)^.

## Current new technology

Recent advancement of DBS device are remarkable. A conventional IPG has a primary cell battery, and IPG replacement surgery is necessary every 4-5 years. Currently, a rechargeable IPG is developed. Rechargeable IPG have a battery life of over 15 years.

MRI was not officially approved for patients with a conventional IPG. However, most current IPGs are MRI compatible under specific conditions of use.

**Figure 4:**
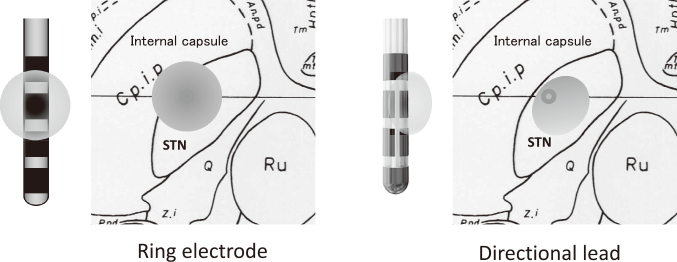
Current steering with directional lead In the conventional ring electrode, increasing amplitude spread stimulation to the internal capsule and induce adverse effect (left). This adverse effect can be avoided by current steering using directional lead (right).

The new technology of the multiple independent current control (MICC) provides independent current settings in eight contacts in one lead^30)^. Amplitude of each contact is freely adjustable regardless of each impedance. Newly developed directional lead is constructed by 4 contact levels like a conventional 4-contact ring electrode, and total of 8 contacts. The middle two contacts consist of 3-segment electrode with each direction of 120 degree, and distal and proximal contacts are conventional ring electrode^31)^. Combination of MICC technology and the directional lead enabled both vertical and horizontal current steering on purposes. Consequently, MICC directional lead is useful to explore more precise control of motor symptoms and also useful to avoid stimulation-induced adverse effect (Figure 4).

**Figure 5:**
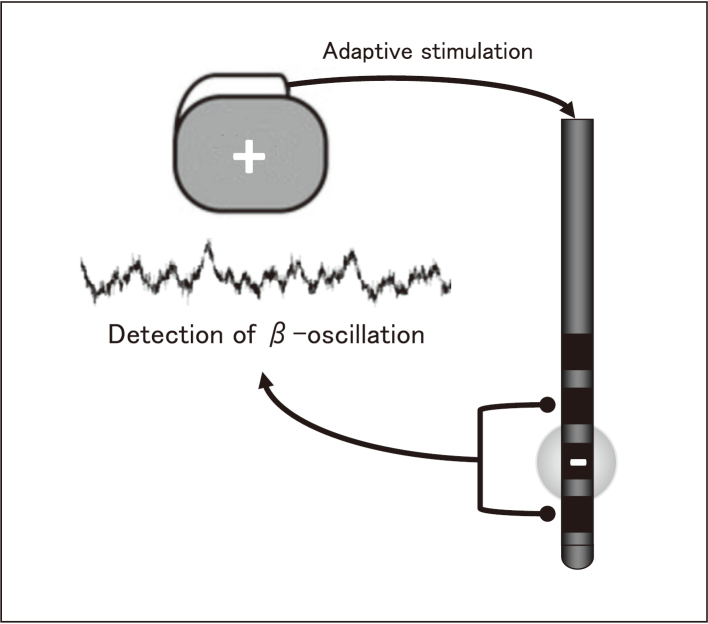
Concept of adaptive DBS

Currently, pathological beta-oscillation recorded from the STN in local field potential recording is the most noteworthy phenomenon in the condition of PD^32)^. The concept of the adaptive DBS is based on a closed-loop model (Figure 5). In adaptive DBS, stimulation amplitude is adjusted according to the detection of pathological beta-oscillation^33)^. The world's first DBS case using commercialized adaptive DBS device was reported from Juntendo Nerima Hospital^34)^. This case demonstrated that the stimulation successfully adapted to beta oscillation fluctuations without any stimulation-induced side effects. This new stimulation method provides new insights into the pathophysiological mechanisms of PD.

## Conclusions

More than twenty years have passed since DBS was introduced in the treatment of PD in Japan. Currently, STN-DBS is the most promising surgical treatment option for patients with medically refractory PD. DBS is also used for other movement disorders and neuropsychiatric diseases. DBS has evolved along with the development of surgical procedures and device technology.

## Funding

No funding was received for this article.

## Author contributions

AU wrote the manuscript.

## Conflicts of interest statement

The Department of Research and Therapeutics for Movement Disorders, Juntendo University Graduate School of Medicine is an endowment department supported with an unrestricted grant from Boston Scientific, Medtronic, Teijin Pharma, Abbvie GK, and FP Pharmaceutical Corporation.
